# Effects of different ventilation modes on intra-abdominal pressure and postoperative nausea and vomiting during anesthesia: a single-blind, randomized controlled trial

**DOI:** 10.3389/fmed.2025.1695970

**Published:** 2026-01-16

**Authors:** Jing Li, Huicong Hu, Li Zhou, Xin Yan, Yaping Lu

**Affiliations:** 1 Anaesthesiology, Zhejiang Chinese Medical University, Hangzhou, Zhejiang, China; 2 Department of Anesthesiology and Pain Medicine, The First Hospital of Jiaxing, Jiaxing, Zhejiang, China; 3 Department of Anesthesiology, HongHui Hospital, Xi’an Jiaotong University, Xi’an, China

**Keywords:** gastric insufflation, facemask ventilation, intra-abdominal pressure, postoperative nausea and vomiting, volume-controlled ventilation

## Abstract

**Background and objectives:**

Improper mask ventilation during anesthesia induction can inject air into the stomach, leading to gastric distension and elevated intra-abdominal pressure (IAP), thereby increasing the risk of reflux and pulmonary aspiration. This single-blind randomized controlled trial compared the effects of three mask ventilation modes—manual ventilation (MV), pressure-controlled ventilation (PCV), and volume-controlled ventilation (VCV)—on IAP during the induction period, and preliminarily observed the dynamic changes of IAP after endotracheal intubation and spontaneous breathing recovery, as well as the incidence of postoperative nausea and vomiting (PONV) in paralyzed patients. We hypothesized that there are differences in the effects of the three mask ventilation modes on IAP during the induction period: PCV may cause less disturbance to IAP than other modes due to its stable pressure control and adjustable tidal volume; meanwhile, IAP will show dynamic changes during the transition from mask ventilation to endotracheal intubation and after the recovery of spontaneous breathing. This hypothesis is based on the physiological mechanism that airway pressure transmission, thoracic-abdominal pressure gradient changes, and gastric distension during mask ventilation may affect IAP.

**Materials and methods:**

A total of 152 participants undergoing laparoscopic surgery were randomized into three ventilation groups, with airway pressure limited to 15 cmH₂O and tidal volume set at 6–8 mL/kg. IAP was measured indirectly via intravesical pressure. The primary outcome was the change in IAP at T0 (before induction) and T1 (after induction of anesthetics) during the mask ventilation period. The secondary outcomes included IAP at T2 (after tracheal intubation) and T3 (24 h after surgery), the incidence of gastric insufflation at T2, the antral cross-sectional area (CSA) at T0 and T2, the incidence and severity of PONV at T3, and hemodynamic and respiratory parameters at each time point.

**Results:**

IAP decreased in all three groups with no significant intergroup differences. However, within the VCV group, patients with gastric insufflation (GI+) showed higher IAP than those without (GI−) (*p* = 0.031). Peak airway pressure was also higher in GI + subgroups in both MV and VCV modes (*p* = 0.009 and *p* < 0.001, respectively). The PCV group exhibited greater delivered tidal volume and lower PaCO₂ (*p* < 0.001). There was no statistically significant difference in the incidence of postoperative nausea and vomiting (PONV) among the three groups, but the incidence of PONV in the gastric insufflation-positive (G+) subgroup was significantly higher than that in the negative (G-) subgroup (25.8% vs. 11.5%, *p* = 0.012). Other secondary outcomes did not differ significantly.

**Conclusion:**

Although all three ventilation modes reduced IAP comparably during the mask ventilation period of anesthesia induction, gastric insufflation was associated with increased IAP within the VCV group. There was no significant difference in the incidence of gastric insufflation or IAP among the three ventilation modes. However, considering that the PCV group had more stable tidal volume delivery and lower PaCO₂, it may have potential advantages in maintaining respiratory stability during the induction period, which needs to be verified by further studies with larger sample sizes.

**Clinical trial registration:**

https://www.chictr.org.cn/showproj.html?proj=208066, identifier (ChiCTR2300076444).

## 1 Introduction

Reflux and aspiration of gastric contents are major concerns for patient safety during general anesthesia and can be fatal. The gastric contents are discharged from the esophagus and pharynx into the oral cavity and then inhaled into the respiratory tract, causing respiratory symptoms or complications, such as aspiration pneumonitis and aspiration pneumonia (1). Aspiration most commonly occurs during the induction of general anesthesia (2). After anesthesia and the application of skeletal muscle relaxants, for patients with apnea who have unprotected airways, improper mask positive pressure ventilation injects air into the stomach, causing gastric volume expansion and increasing the patient’s risk of reflux and aspiration, especially for patients who are full or whose ability to protect the airway is impaired (3, 4).

The association between pulmonary aspiration and gastric aspiration also depends on other factors that promote reflux of gastric contents, such as intra-abdominal pressure (IAP) (5). The mechanisms of reflux include temporary complete relaxation of the lower esophageal sphincter, a temporary increase in the IAP, or a decrease in the resting pressure of the lower esophageal sphincter (6). Intravesical pressure measurement is considered the gold standard for assessing IAP (7). Relevant studies have shown that intragastric pressure, intravesical pressure, and the IAP are strongly correlated (8). Under general anesthesia, lower esophageal sphincter barrier pressure decreases significantly and approaches zero in some anaesthetized patients, suggesting that reflux aspiration may occur even with a small increase in intragastric pressure (9).

The use of appropriate mask ventilation patterns during anesthesia induction can ensure adequate ventilation and reduce the risk of air entering the stomach during face-mask ventilation (10). Manual ventilation (MV) is a core component of airway management (11). Volume-controlled ventilation (VCV) ensures target ventilation via constant flow but may result in high peak airway pressure (12). On the other hand, in pressure-controlled ventilation (PCV) mode, the ventilator can provide constant pressure, avoiding excessive peak airway pressures (13).

### 1.1 Study hypothesis and theoretical basis

Based on the existing literature, we propose the following research hypothesis: There are differences in the effects of three mask ventilation modes (MV, PCV, and VCV) on IAP during the induction period. PCV may cause less disturbance to IAP than MV and VCV because it can maintain stable inspiratory pressure and adjust tidal volume more flexibly; meanwhile, IAP will show dynamic changes during the transition from mask ventilation to endotracheal intubation and after the recovery of spontaneous breathing. The theoretical basis is as follows: (1) airway pressure during mask ventilation can be transmitted to the abdominal cavity through the thoracic cavity, affecting IAP; (2) different ventilation modes have different effects on peak airway pressure and tidal volume, and PCV can avoid excessive peak pressure compared with VCV; (3) neuromuscular blockers used during induction can relax abdominal muscles and reduce baseline IAP, which may interact with the effect of the ventilation mode on IAP; and (4) gastric insufflation caused by mask ventilation may increase intragastric pressure and further affect IAP.

In the current literature, ventilation with 15 cmH₂O pressure or 6–8 mL/kg tidal volume during anesthesia induction reduces gastric insufflation, with PCV often considered preferable (14, 15). However, changes in IAP under different ventilation modes during induction remain unclear. Thus, this single-blind randomized controlled trial aimed to compare the effects of MV, PCV, and VCV on IAP during induction (primary outcome: T0-T1 IAP change). Secondary aims included observing dynamic IAP changes after intubation and spontaneous breathing recovery, and assessing gastric insufflation and PONV incidence. We hypothesized that PCV causes less IAP disturbance than MV/VCV, and IAP dynamically changes during ventilation transitions.

## 2 Materials and methods

### 2.1 Study design and ethics

This prospective randomized study was approved by the Ethics Committee of Jiaxing First Hospital (approval number: 2023-LY-451) and was performed at the Department of Anesthesiology from October 2023 to April 2024. The protocol was registered in the Chinese Clinical Trial Registry at www.chictr.org.cn (trial number: ChiCTR2300076444). The present manuscript was written in adherence with the Consolidated Standard of Reporting Trials (CONSORT) guidelines (16).

### 2.2 Participants

After providing written informed consent, 162 patients aged 18–75 years with ASA grades ranging from I–III, and who were scheduled for preoperative catheterization and laparoscopic surgery, were enrolled in the study. We excluded patients who were pregnant, had undergone stomach surgery, were unable to perform semi-recumbent decubitus (SRD) position, had previously undergone upper gastrointestinal and urological surgery, had difficult airways, had severe circulatory and respiratory diseases, or had diseases affecting the IAP measurement.

### 2.3 Definition and assessment method of gastric insufflation (G+/−) subgroups

In this study, the gastric insufflation-positive subgroup (G+) and gastric insufflation-negative subgroup (G-) were defined based on the results of antral ultrasound examination. The specific assessment tool used was a GE Logiq E9 color Doppler ultrasound diagnostic instrument (probe model: 3.5–5.0 MHz convex array probe). The assessment was performed at T1. Patients were placed in the supine position, and the probe was placed at the midline below the xiphoid process, slightly to the right. After clearly displaying the short-axis view of the gastric antrum, the anteroposterior diameter (AP) and craniocaudal diameter (CC) of the antrum were measured, and the gastric antral cross-sectional area (GCSA) was calculated using the formula: GCSA = *π* × (AP/2) × (CC/2). Referring to the criteria in the “Expert Consensus on Perioperative Ultrasound Assessment of Gastric Contents” (2023 Edition), GCSA > 340 mm^2^ was defined as gastric insufflation-positive (G+), and GCSA ≤ 340 mm^2^ was defined as gastric insufflation-negative (G-). All ultrasound examinations were independently performed by two physicians with more than 5 years of experience in anesthetic ultrasound. If the difference between the two measurement results exceeded 10%, a third senior physician reviewed and determined the final result to ensure the consistency of the assessment.

### 2.4 Randomization and blinding

**Figure 1:**
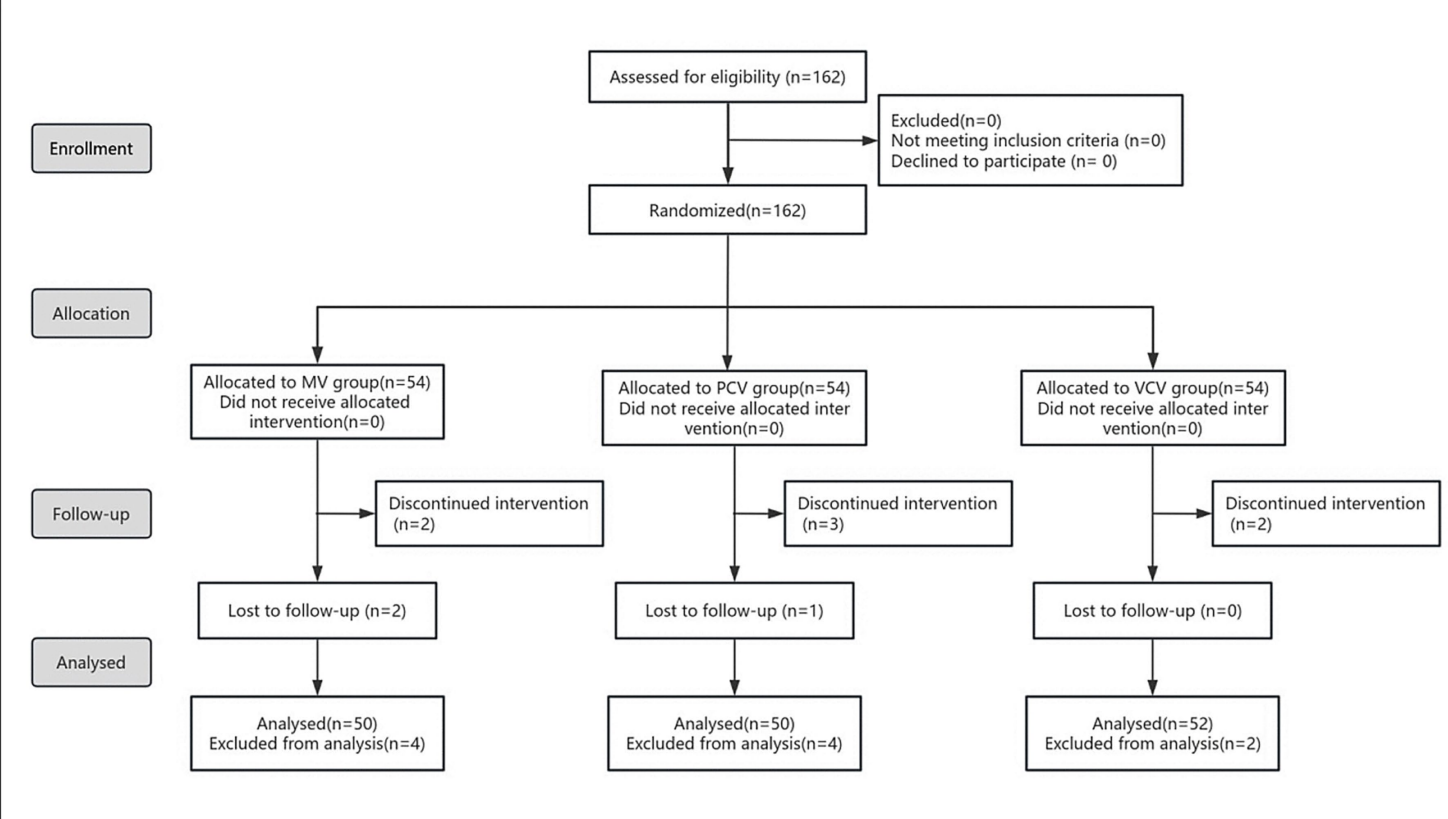
CONSORT flow diagram. MV, manual ventilation; VCV, volume-controlled ventilation; PCV, pressure-controlled ventilation.

The participants were randomized via a computer-generated list of random numbers to allocate patients in a 1:1:1 ratio to the MV, PCV, and VCV groups. The attending anesthesiologists were aware of the group assignment, but the patients, outcome investigators, and data analysts were blinded to the allocated group. The enrollment and allocation of patients are summarized in a CONSORT flow diagram (Figure 1).

### 2.5 Ventilation protocol

The same type of mechanical ventilator (Datex-Ohmeda-Avance anesthesia machine, GE Healthcare, Madison, United States) was selected, and pre-use leakage and compliance tests were performed before the start of anesthesia. In the MV group, the APL valve was set to 15 cmH_2_O, the V_T_ was 6–8 mL/kg, and the frequency was 12 times/min (according to the metronome). In the PCV group, the pressure was set to *p* = 15 cmH_2_O, and the respiratory rate was 12 breaths/min (15). In the VCV group, the VT was set at 6–8 mL/kg, and the respiratory rate was 12 breaths/min. All three research groups were set to have no positive end-expiratory pressure (PEEP), a respiratory rate of 12, an inhalation-to-exhalation ratio of 1:2, and 100% oxygen for 180 s.

### 2.6 Procedures

After the patient entered the operating room and rested for 15 min (T0), a routine anesthesia monitor was used to monitor the heart rate (HR) and peripheral capillary oxygen saturation (SpO_2_). The radial artery was cannulated to measure the mean arterial pressure (MAP), and arterial blood gas measurements were performed with a blood gas analyzer (ABL-800 FLEX series, Radiometer, Bronshoj, Denmark). The antral cross-sectional area (CSA) was measured by ultrasound when the patient was in the SRD position. A Foley catheter was inserted into the patient, and the IAP was measured indirectly while the patient was in the supine position.

All patients received preoxygenation therapy (100% flow rate of 8 L/min, 3 min), intravenous injections of atropine (0.01 mg/kg), propofol (2 mg/kg), rocuronium bromide (0.6 mg/kg), and sufentanil (0.3 μg/kg) for anesthesia induction. After the patient lost consciousness (T1), the IAP was recorded. The same assistant started the two-handed mask ventilation method, and the face mask ventilation method was divided into MV, PCV, or VCV according to the grouping. The trachea was intubated after 3 min of mask ventilation. After tracheal intubation (T2), the IAP and vital signs were measured, and the antral CSA was measured in the SRD position.

The patients were subsequently connected to a mechanical ventilator with a 60% fraction of inspired O_2_, and an end-tidal carbon dioxide of 35–45 mmHg was maintained. Anesthesia was maintained with sevoflurane to maintain the bispectral index between 40 and 60, and remifentanil was administered at 0.05–0.3 μg/kg/min with a pump (double-channel microinfusion pump WZS-50F6; Smiths Medical Instrument Co., Ltd., Zhejiang, China) to maintain hemodynamic stability. Boluses of 0.03 mg/kg cisatracurium were intermittently injected to maintain muscle relaxation. After the operation, the patient’s tracheal catheter was removed after it met the extubation standard.

Before the end of the operation, the patient was given 0.1 mg/kg ondansetron to prevent PONV. Each patient received intravenous patient-controlled analgesia (IV-PCA) after surgery. IV-PCA was prepared with 1.5 μg/kg sufentanil and 8 mg ondansetron in 0.9% normal saline for a total volume of 100 mL. The IV-PCA device (Rehn Med Tech Ltd., Jiangsu, China) was connected at the end of surgery (2 mL demand dose, 20 min lockout, 2 mL/h background infusion). Postoperatively, the patient was transferred to the ward. Twenty-four hours after surgery (T3), PONV was recorded according to the simplified PONV impact scale of Myles et al.

#### 2.6.1 Data collection methods

In the study, the manometer tube was connected to the Foley urinary catheter, 25 mL of 0.9% normal saline was injected into the bladder, the pubic symphysis was used as the zero plane, the height of the water column was read at the end of expiration, and the measured value was the IAP.

The gastric antrum CSA was examined via the high-frequency probe of an ultrasound monitor (SC6-1 U/Resona7, Mindray, Shenzhen, China), with the patient in the SRD position. The gastric antrum image was obtained in the sagittal or parasagittal plane between the left lobe of the liver and the pancreas at the level of the aorta or inferior vena cava. Gastric insufflation was defined by an acoustic shadow phenomenon and/or a comet-tail artifact in the antrum on ultrasonography. The longitudinal (D1) and anteroposterior (D2) diameters of the gastric antrum were determined. The antral CSA was calculated as follows, assuming that the antrum had an elliptical shape: CSA = (D1 × D2 × *π*)/4.

Documentation and assessment of postoperative nausea and vomiting were performed according to the simplified PONV impact scale of Myles et al. (17). This evaluation scale includes two questions: Q1. Have you vomited or had dryretching? Q2. Have you experienced nausea?. The maximum total score of the evaluation scale is 6 points, and the minimum score is 0 points.

### 2.7 Outcomes

The primary outcome was the change of IAP at T0 (15 min after entering the operating room, before induction) and T1 (after intravenous injection of anesthesia-inducing drugs, during mask ventilation) to reflect the effect of different mask ventilation modes on IAP during the induction period.

The secondary outcomes included:

1. IAP at T2 (after tracheal intubation) and T3 (24 h after surgery) to observe the dynamic changes of IAP;
2. The incidence of gastric insufflation at T2;
3. The antrum CSA at T0 and T2;
4. The incidence and severity of PONV at T3;
5. Hemodynamic parameters (HR, MAP) at T0 and T2;
6. Respiratory and ventilatory variables (peak airway pressure [PAP], VT, SpO_2_, arterial partial pressure of oxygen [PaO_2_], and partial pressure of carbon dioxide [PaCO_2_]) at T0 and T2.

The data were recorded at the following time points by trained doctors.

- T0: 15 min after entering the operating room.
- T1: after intravenous injection of anesthesia-inducing drugs.
- T2: after tracheal intubation.
- T3: 24 h after surgery.

### 2.8 Statistical analysis

On the basis of a pilot study of 15 patients, a population similar to that in our current study, with abdominal pressure values measured after tracheal intubation, the mean values for the three groups were 6.8, 5.9, and 7.6, respectively, with standard deviations of 2.1, 1.7, and 2.5. One-way analysis of variance revealed that a total of 129 patients were needed to achieve a two-sided alpha significance level of 0.05 and a statistical power of 80%. Taking into account a 20% dropout rate, we estimated that a total of 162 patients would be needed, with 54 in each group.

Data are summarized by the mean (standard deviation), median [interquartile range (IQR)], or number (%). The normality of the continuous variable distribution was assessed via the Shapiro–Wilk W test, and Levene’s test was used to evaluate the equality of variances for a variable. Categorical variables are summarized as numbers and were analyzed via the chi-squared test.

A post-hoc power analysis was performed to verify the adequacy of power for the primary outcome (T0-T1 IAP change), as the initial sample size calculation was based on T2 IAP data due to limited pilot data on T0-T1 changes at the study design stage. Using G*Power 3.1.9.7 software, we inputted the observed mean IAP changes (MV group: 1.79 ± 2.05 cmH₂O; PCV group: 1.63 ± 2.11 cmH_2_O; VCV group: 1.91 ± 2.23 cmH_2_O) and standard deviations (MV: 2.39, PCV: 2.45, VCV: 2.57) from the study. With a sample size of 152 patients, *α* = 0.05, and a two-tailed test, the post-hoc power was calculated as 82.3%, which exceeds the conventional threshold of 80%, indicating that the study was adequately powered for the primary outcome.

All the statistical data were analyzed via SPSS software version 23.0(IBM Corporation, Armonk, New York, United States). The study had three different modes of ventilation. Data at each time point with a normal distribution and equal variance were compared via one-way analysis of variance (ANOVA) with the Bonferroni *post hoc* correction. For continuous variables with two or more nonnormally distributed variables in multiple groups, the Kruskal–Wallis test was used to compare multiple groups. Comparisons of variables over different time points within groups were accomplished via repeated-measures ANOVA or the Friedman test on the basis of the normality of the variable’s distribution. Statistical significance in the evaluation was determined by a *p* value less than 0.05. Nevertheless, a correction of multiple comparisons was conducted.

## 3 Results

### 3.1 Study population

A total of 162 patients received qualified screenings and were randomly assigned to one of the study groups. There were four patients in the MV group, four patients in the PCV group, and two patients in the VCV group; these patients dropped out because IAP measurement failure did not occur, the display of the gastric antrum was unclear, and data were lost. Therefore, 152 patients were eligible for the final analysis (Figure 1). The demographic and baseline characteristics of the patients in the three study groups were comparable (Table 1).

**Table 1:** Baseline characteristics of study subjects.

Variables	MV group (*n* = 50)	PCV group (*n* = 50)	VCV group (*n* = 52)	*P*-value
Age (years)	60.50 ± 9.28	58.28 ± 10.16	57.44 ± 11.26	0.305
BMI (kg/m^2^)	23.08 ± 3.05	23.60 ± 2.90	23.31 ± 2.70	0.665
Male sex	21 (42.0%)	29 (58.0%)	27 (51.9%)	0.270
Smoking	9 (18.0%)	11 (22.0%)	8 (15.4%)	0.687
Drink alcohol	8 (16.0%)	9 (18.0%)	6 (11.5%)	0.646
Hypertension	16 (32.0%)	18 (36.0%)	19 (36.5%)	0.872
Diabetes mellitus	7 (14.0%)	3 (6.0%)	5 (9.6%)	0.412
ASA grade				0.177
II	34 (68.0%)	41 (82.0%)	35 (67.3%)	
III	16 (32.0%)	9 (18.0%)	17 (32.7%)	
Fasting time (h)	9 (9.13)	10 (8, 12.5)	10.5 (9.13)	0.771
Surgical time (h)	2 (2.3)	2.5 (2, 3.5)	2.75 (2.3)	0.173
Surgical procedure				0.591
Colon	22 (44.0%)	22 (44.0%)	23 (44.2%)	
Rectum	14 (28.0%)	9 (18.0%)	9 (17.3%)	
Kidney	10 (20.0%)	9 (18.0%)	10 (19.2%)	
Liver	4 (8.0%)	10 (20.0%)	10 (19.2%)	
Title of anesthesiologist				0.335
Chief physician	7 (14.0%)	11 (22.0%)	15 (28.8%)	
Associate chief physician	14 (28.0%)	12 (24.0%)	8 (15.4%)	
Attending physician	29 (58.0%)	27 (54.0%)	29 (55.8%)	

The data are presented as the means ± SDs or medians with IQRs or frequencies (%). MV, manual ventilation; PCV, pressure-controlled ventilation; VCV, volume-controlled ventilation; BMI, body mass index; ASA, American Standards Association.

### 3.2 Primary outcome: intra-abdominal pressure and incidence of gastric insufflation

**Figure 2:**
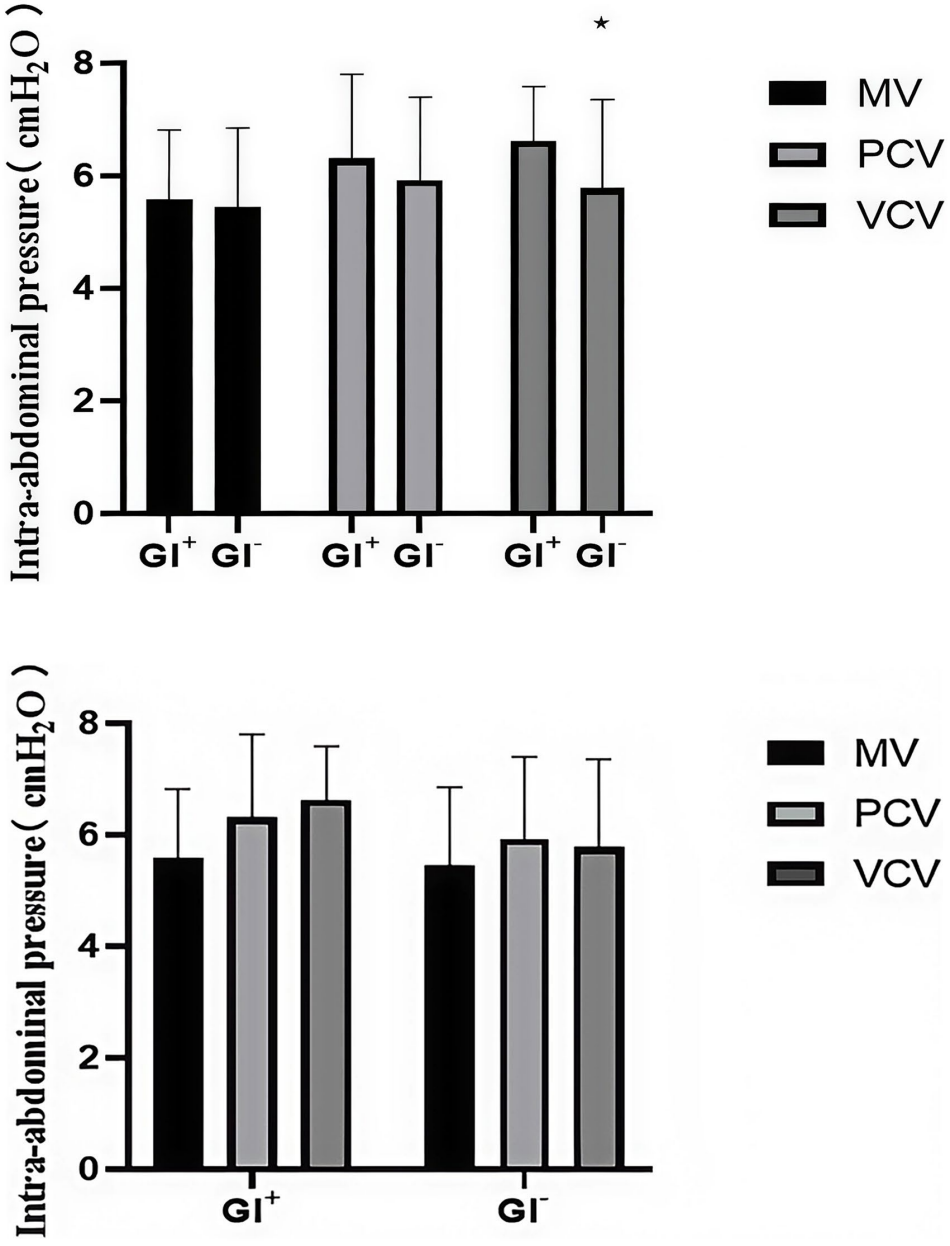
Intra-abdominal pressure (IAP) of the three groups in the GI + subgroup and the GI − subgroup at T3. GI + = gastric insufflation detected by ultrasonography; GI − = gastric insufflation not detected by ultrasonography. *p < 0.05 versus the GI + subgroup in each group at T3 was studied.

The primary outcome of this study was the change in IAP at T0 (before induction) and T1 (during mask ventilation) to evaluate the effect of different mask ventilation modes. In terms of the main results, we observed that the IAP in all three groups at T1 was significantly lower than that at T0 (*p* < 0.001), indicating that all three mask ventilation modes were associated with a decrease in IAP during the induction period. There was no significant difference in IAP between the three groups at T0 and T1 (Table 2). At three time points, there was no significant difference in the IAP among the three groups (Table 2). Further subgroup analysis showed that at T2, there was no significant difference in IAP among the three groups in either the GI + or GI − subgroup. However, within the VCV group, the IAP of the GI + subgroup was significantly higher than that of the GI − subgroup (*p* = 0.031) (Table 3), while there was no significant difference between the GI + and GI − subgroups in the MV and PCV groups (Figure 2).

**Table 2:** IAP and incidence of gastric insufflation before and after the anesthesia induction period.

Variables	MV group (*n* = 50)	PCV group (*n* = 50)	VCV group (*n* = 52)	*P*-value
GI^+^	9 (18.0%)	16 (32%)	12 (23.1%)	0.255
IAP (cmH_2_O)				
T0	8.83 ± 2.67	9.39 ± 2.72	9.10 ± 2.88	0.596
T1	7.04 ± 2.12	7.76 ± 2.18	7.19 ± 2.26	0.228
T2	5.48 ± 1.36	6.05 ± 1.47	5.96 ± 1.50	0.108

**Table 3:** Iap comparison between GI + and GI − subgroups across three ventilation modes.

Ventilation mode	Subgroup	Sample size (n)	IAP (cmH_2_O, mean ± SD)	*P*-value (subgroup comparison within mode)	*P*-value (intermode comparison within subgroup)
MV	GI+	9	5.89 ± 1.42	0.387	0.215
	GI−	41	5.37 ± 1.34		
PCV	GI+	16	6.23 ± 1.51	0.452	0.189
	GI−	34	5.92 ± 1.45		
VCV	GI+	12	6.57 ± 1.55	0.031	0.098
	GI−	40	5.68 ± 1.41		

The data are presented as the means ± SDs or frequencies (%). MV, manual ventilation; PCV, pressure-controlled ventilation; VCV, volume-controlled ventilation; GI^+^, gastric insufflation detected by ultrasonography; IAP, intra-abdominal pressure.

A standardized postoperative diet management protocol was adopted in this study: all patients fasted and abstained from water within 6 h after surgery, and only a clear liquid diet (such as warm boiled water and rice soup) was given within 6–24 h after surgery, with the intake controlled within 500 mL. The dietary intake was uniformly recorded by research nurses to ensure the consistency of diet management among the groups. Subgroup analysis showed that there was no statistically significant difference in dietary intake at 24 h postoperatively between the G + and G- subgroups (*p* = 0.892), indicating that dietary factors did not interfere with the IAP comparison at Time Point T3.

### 3.3 Variables before and after the anesthesia induction period

After anesthesia induction, the heart rate was significantly lower at T1 and T2 than at T0 in all three groups (*p* < 0.001 for all), and the MAP was the highest at T0 and the lowest at T2 in all three groups (*p* < 0.001 for all). For the respiratory and ventilation variables, SPO_2_ was significantly greater at T2 than at T0 in all three groups (*p* < 0.001, *p* < 0.001, and *p* = 0.002), and PaO_2_ was significantly greater at T2 than at T0 in all three groups (*p* < 0.001 for all). PaCO_2_ in the MV group and the VCV group was significantly greater at T2 than at T0 (*p* < 0.001 and *p* = 0.002), the MV group had greater PaCO_2_, and the PCV group had lower PaCO_2_ at T2 (*p* < 0.001). The antral CSA in the VCV group was significantly greater at T2 than at T0 (*p* = 0.043). Other respiratory and ventilation variables, hemodynamic parameters, and antral CSA were comparable among the groups (Table 4).

**Table 4:** Changes in the observation indices before and after the anesthesia induction period.

Variables	Time	MV group	PCV group	VCV group	95% CI	*P*-value
HR (bpm)	T0	80.16 ± 11.61	76.36 ± 11.10	81.52 ± 13.45	[77.38,81.27]	0.094
	T2	73.58 ± 12.52^*^	70.98 ± 11.48^*^	73.86 ± 15.06^*^	[70.74,74.92]	0.474
MAP (mmHg)	T0	108.42 ± 11.95	104.54 ± 13.20	108.54 ± 11.90	[104.90,108.93]	0.262
	T2	83.54 ± 14.29^*^	79.12 ± 12.53^*^	82.90 ± 11.0.33^*^	[79.65,83.75]	0.196
SPO_2_ (%)	T0	96 (95.98)	97 (95.98)	97 (95.99)	[96.28,97.02]	0.283
	T2	99 (98,100)^*^	99 (97.75,100)^*^	99 (97,100)^*^	[98.08,98.68]	0.628
PaO_2_ (mmHg)	T0	83.70 ± 10.02	85.86 ± 9.42	85.46 ± 9.06	[83.43,86.46]	0.500
	T2	228.04 ± 58.65^*^	242.38 ± 59.52^*^	234.64 ± 55.64^*^	[225.76,244.31]	0.466
PaCO_2_ (mmHg)	T0	39.04 ± 1.88	38.22 ± 2.57	38.86 ± 1.60	[38.36,39.03]	0.121
	T2	42.08 ± 2.77^*†^	37.14 ± 3.46^†‡^	40.18 ± 2.50^*†‡^	[39.23,40.36]	<0.001
CSA (mm^2^)	T0	329.24 ± 88.31	348.48 ± 82.20	359.72 ± 87.15	[329.54,357.69]	0.360
	T2	351.02 ± 91.04	374.16 ± 123.72	403.62 ± 107.45^*^	[355.70,391.42]	0.142

The data are presented as the means ± SDs or medians with IQRs. CI, confidence interval; MV, manual ventilation; PCV, pressure-controlled ventilation; VCV, volume-controlled ventilation; HR, heart rate; MAP, mean arterial pressure; SPO_2_, pulse oxygen saturation; PaO_2_, arterial partial pressure of oxygen; PaCO_2_, partial pressure of carbon dioxide; CSA, cross-sectional area. ^*^p < 0.05 vs. T0 in each group. ^†^*p* < 0.05 for the MV group vs. the PCV group or the MV group vs. the VCV group at the same time point. ^‡^*p* < 0.05 for the PCV group vs. the VCV group at the same time point.

### 3.4 Peak airway pressure and tidal volume

**Figure 3:**
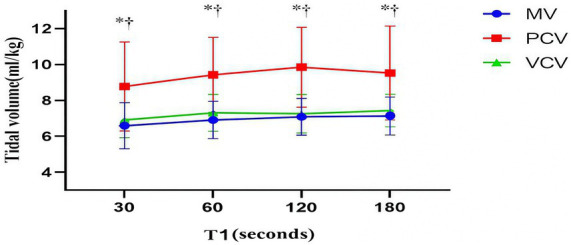
Tidal volume. The markers are the means, and the error bars are the standard deviations. MV, manual ventilation; PCV, pressure-controlled ventilation; VCV, volume-controlled ventilation. *p < 0.05 for the PCV group vs. the MV group at the same time point. †*p* < 0.05 for the PCV group vs. the VCV group at the same time point.

In the GI^−^ subgroup, the PAP of the PCV group was greater than that of the MV group and the VCV group (*p* < 0.001). In the GI^+^ subgroup, there was no significant difference in PAP among the three groups. In the GI^−^ and GI^+^ subgroups, the V_T_ of the PCV group was greater than that of the MV and VCV groups (*p* < 0.001) (Table 5). During the mask ventilation process, the V_T_ of the PCV group was significantly greater than that of the MV group and VCV group (*p* < 0.001 for all) (Figure 3).

**Table 5:** Tidal volume and peak airway pressure during mask ventilation.

Group	Variables	MV group (*n* = 50)	PCV group (*n* = 50)	VCV group (*n* = 52)	*P*-value
GI^+^	V_T_ (mL/kg)	6.73 ± 0.86^†^	9.06 ± 2.01^†^	6.99 ± 0.83^†^	<0.001
	PAP (cmH_2_O)	14 (13.16)	15 (15.15)	16 (15.18)	0.056
GI^−^	V_T_ (mL/kg)	7.49 ± 0.90^†^	7.61 ± 1.48^†^	7.48 ± 0.74^†^	<0.001
	PAP (cmH_2_O)	13 (12.14)^*†^	15 (15.15)^†^	13 (12.15)^*†^	<0.001

The data are presented as the means ± SDs or medians with IQRs. MV, manual ventilation; PCV, pressure-controlled ventilation; VCV, volume-controlled ventilation; V_T_, tidal volume; PAP, peak airway pressure; GI^+^, gastric insufflation detected by ultrasonography; GI^−^, gastric insufflation not detected by ultrasonography. *p < 0.05 vs. GI^+^ in each group. ^†^*p* < 0.05 for the PCV group vs. the MV group or the PCV group vs. the VCV group in the GI^+^ or GI^−^ subgroups.

### 3.5 Postoperative nausea and vomiting

Regarding the assessment of PONV, we found that 30.0% of patients in the MV group, 34.0% of patients in the PCV group, and 34.6% of patients in the VCV group met the primary endpoint of experiencing PONV. However, after statistical analysis, the three groups showed no statistically significant differences in the incidence of nausea and vomiting or the severity of PONV within 24 h after surgery (Table 6).

**Table 6:** Incidence and severity of postoperative nausea and vomiting.

Variables	MV group	PCV group	VCV group	*P*-value
Nausea	15 (30.0%)	17 (34.0%)	18 (34.6%)	0.866
Vomiting	6 (12.0%)	9 (18.0%)	5 (9.6%)	0.437
PONV severity				
0	35 (70.0%)	32 (64.0%)	34 (65.4%)	0.585
1	8 (16.0%)	8 (16.0%)	12 (23.1%)	
2	5 (10.0%)	6 (12.0%)	2 (3.8%)	
3	1 (2.0%)	2 (4.0%)	2 (3.8%)	
4	0 (0.0%)	2 (4.0%)	0 (0.0%)	
5	1 (2.0%)	0 (0.0%)	2 (3.8%)	
6	0 (0.0%)	0 (0.0%)	0 (0.0%)	

The data are summarized by number (%). MV, manual ventilation; PCV, pressure-controlled ventilation; VCV, volume-controlled ventilation; PONV, postoperative nausea and vomiting. PONV Severity uses the simplified PONV impact scale proposed by Myles et al. This scale uses a six-point scoring system, where the highest score of 6 points represents the most severe PONV symptoms, whereas the lowest score of 0 represents no PONV symptoms.

There was no statistically significant difference in the overall incidence of PONV among the three groups. However, subgroup analysis showed that the incidence of PONV in the G + subgroup was significantly higher than that in the G- subgroup among all patients (25.8% vs. 11.5%, *p* = 0.012). Stratified analysis showed that the incidence of PONV in the G + subgroup of the VCV group was the highest (33.3%), which was significantly higher than that in the G- subgroup of the same group (10.0%, *p* = 0.021). There were no statistically significant differences in the incidence of PONV between the G + and G- subgroups in the MV group (22.2% vs. 15.0%, *p* = 0.523) and the PCV group (23.5%vs. 11.8%, *p* = 0.367).

## 4 Discussion

Gastric insufflation during anesthesia induction is a common clinical issue that may be associated with increased intra-abdominal pressure (IAP) and an elevated risk of postoperative complications like postoperative nausea and vomiting (PONV). Different ventilation strategies [manual ventilation (MV), pressure-controlled ventilation (PCV), and volume-controlled ventilation (VCV)] applied during induction may exert varying effects on gastric insufflation and subsequent IAP changes, yet relevant comparative evidence remains insufficient. The core objectives of this study were (1) to primarily evaluate the impact of MV, PCV, and VCV modes on IAP during anesthesia induction; (2) to secondarily explore the differential effects of these ventilation modes on IAP in patients with or without gastric insufflation (G+/− subgroups), analyze the association between gastric insufflation and PONV, and observe the dynamic changes of IAP up to 24 h postoperatively.

In this randomized controlled trial of patients undergoing laparoscopic surgery, we observed changes in the IAP in different ventilation modes during the anesthesia induction period to explore the relationships between the IAP and gastric insufflation and PONV. We observed that the IAP decreased after mask ventilation, that there was no significant difference among the three groups, and that the IAP of the GI^+^ subgroup in the VCV group was greater than that of the GI^−^ subgroup. There was no significant difference in the incidence of gastric insufflation among the three groups. The MV and VCV groups presented higher peak airway pressures than did the GI^+^ subgroup. In addition, the PCV group presented greater V_T_ and lower PaCO_2_. No statistically significant differences in the other study parameters were detected among the three ventilation modes.

We selected three modes of ventilation: manual ventilation, pressure-controlled ventilation, and volume-controlled ventilation. The VCV mode maintains a constant flow rate during ventilation, but may cause a linear increase in airway pressure (18). PCV mode relies on preset inspiratory pressure to work. However, this mode does not ensure the consistency of V_T_ because any change in lung or chest compliance may affect the delivery of V_T_ (19, 20). In contrast, the manual ventilation group tended to exhibit a wider range of changes in peak airway pressure and V_T_ during surgery (10). According to Bouvet et al., in patients who are not obese and have not received muscle paralysis treatment, pressure-controlled ventilation using 15 cmH_2_O can effectively reduce gastric insufflation (15). In this study, an inspiratory pressure of 15 cmH_2_O was selected, and the corresponding APL threshold and V_T_ of 6–8 mL/kg were set.

In previous studies, ultrasound technology was often used to assess the gastric residual volume to predict the risk of gastric aeration and pulmonary aspiration (21, 22). An increase in gastric volume is usually accompanied by an increase in gastric pressure (23). Previous studies have confirmed the close correlation between intragastric pressure and IAP (8, 24). To protect the anatomical integrity of the upper esophageal sphincter and lower esophageal sphincter, we rejected direct invasive pressure measurement to monitor intragastric pressure (25). We used cystometry as an indirect means to assess the IAP.

In this study, we observed that the IAP decreased after mask ventilation, and there was no significant difference among the three groups; however, all three groups had gastric insufflation caused by a comet-tail artifact in the antrum on ultrasonography. The results of this study contrast with those of S von Delius, who reported that gastric aeration causes a significant increase in IAP (26). Our initial hypothesis that PCV would cause less IAP disturbance was not supported by the primary outcome (no significant intergroup difference in IAP change from T0 to T1). We propose that this discrepancy is due to the dominant IAP-lowering effect of neuromuscular blockers, which may have offset the potential IAP-disturbing effects of different ventilation modes. This explanation is consistent with previous studies that confirmed neuromuscular blockers can significantly reduce baseline IAP by relaxing abdominal muscles. Neuromuscular blocking agents have been shown to reduce the IAP (27, 28). Although the primary IAP outcome failed to show intergroup differences, the secondary outcomes still provide valuable clinical insights. The PCV group exhibited more stable delivered tidal volume and lower PaCO₂ (*p* < 0.001) compared with MV and VCV groups, which reflects PCV’s advantage in maintaining respiratory stability during anesthesia induction—a key clinical goal to avoid hypoxemia and hypercapnia. These findings do not negate the primary outcome but complement it by revealing PCV’s potential value in respiratory management. Future studies with larger sample sizes are needed to verify whether these respiratory advantages can translate into reduced incidence of postoperative respiratory complications. We also analyzed the IAP and PAP of the GI^+^ subgroup and the GI^−^ subgroup. In the GI^+^ subgroup, the MV and PCV groups presented greater PAP, but there was no significant difference in IAP between the GI^+^ subgroup and the GI^−^ subgroup. We believe that even if the PAP is too high, it will not necessarily affect the IAP.

In the study design, the peak airway pressure or tidal volume of the three groups of ventilation modes we selected were within a certain range. We chose an inspiratory pressure of 15 cmH_2_O, and the corresponding APL threshold and V_T_ were set to 6–8 mL/kg, and the V_T_ and PAP during mask ventilation were recorded. In this study, the V_T_ of the PCV group was significantly greater than that of the MV group and the VCV group. The results of Ulrich Goebel’s study are similar to those of this study; while equal V_T_ was maintained, the PCV group presented a lower peak airway pressure than did the MV group (29). However, since there was no significant difference in peak airway pressure among the three groups, there was no difference in the incidence of gastric aeration among the three groups.

Previous studies reported that the VT in the PCV group was much greater than 6–8 mL/kg, but there was no obvious gastric insufflation (30). We believe that gastric insufflation is related mainly to the PAP rather than the V_T_. We found that the incidence of gastric insufflation with 15 cmH_2_O pressure-controlled ventilation was the highest among the three groups. Some studies have compared different inspiratory pressures and have shown that the incidence of gastric insufflation is lower with an inspiratory pressure of 10 cmH_2_O (30). Related studies have reported that the PCV mode can improve oxygenation and significantly reduce peak inspiratory pressure (31). Recently, the pressure-controlled ventilation-volume guaranteed (PCV-VG) model has been highly praised, and the PCV-VG model is worth studying (32). During the induction period of anesthesia, pressure-controlled ventilation with lower airway pressure can be considered.

With respect to PONV, there was no significant difference in the overall incidence of PONV among groups in this study, but the overall incidence in the G + subgroup was significantly higher than that in the G- subgroup, and the G + subgroup of the VCV group had the highest PONV risk. This result has important clinical implications. Gastric insufflation may stimulate gastrointestinal stretch receptors and activate the vomiting reflex pathway, thereby increasing the risk of PONV. The more significant increase in IAP in the G + subgroup under the VCV mode may further aggravate this stimulating effect. This finding suggests that for patients with gastric insufflation during anesthesia induction, especially those using the VCV mode, preventive measures for PONV should be strengthened after surgery, such as the prophylactic use of 5-hydroxytryptamine receptor antagonists, to improve the postoperative comfort of patients (33, 34).

This study has several limitations. First, the three groups of ventilation modes used similar V_T_ and airway pressures, which may have resulted in no difference in IAP. Second, indirect measurement of the IAP to evaluate gastric insufflation may ignore small changes in the IAP. Finally, the sample size of this study is relatively small, which may affect the accuracy and reliability of the study results to a certain extent.

## 5 Conclusion

In conclusion, when the inspiratory pressure was 15 cmH_2_O and the V_T_ was 6–8 mL/kg, the IAP of all three groups decreased during mask ventilation, but there was no significant difference in the IAP. Excessive gastric insufflation may affect the IAP. During the induction period of anesthesia, pressure-controlled ventilation with lower airway pressure can be considered. The conclusion of our study indeed “deviated” from the general expectation. Although this finding seems contrary to traditional cognition at first glance, we believe it precisely reveals the complex interaction of multiple physiological factors in clinical practice. We propose a possible explanation for this phenomenon that requires further verification, focusing on the dominant physiological effect: we hypothesize that the relaxation of systemic skeletal muscles (especially abdominal wall muscles) induced by anesthetics and muscle relaxants exerts a dominant and overwhelming effect on reducing IAP. In contrast, under standardized operation, the potential pressure-increasing effect caused by a small amount of gas entering the stomach may be masked by this strong dominant effect, thus still resulting in a net decrease in IAP. Therefore, we believe that the data of this study do not refute the classic theory, but rather provide important supplementation and contextual limitations to it. Our findings suggest that under strictly standardized operation, the risk of IAP increase caused by mask ventilation may be controllable, and its net effect may even manifest as a decrease. Certainly, this is only the finding of a single study, and its generalizability needs to be confirmed by larger-scale studies.

## Data Availability

The raw data supporting the conclusions of this article will be made available by the authors, without undue reservation.
